# Supplementary material for: The interactome of intact mitochondria by cross-linking mass spectrometry provides evidence for coexisting respiratory supercomplexes
Source: Mol Cell Proteomics. 2017 Dec 8;17(2):216–32. doi: 10.1074/mcp.RA117.000470 (PMC5795388; doi:10.1074/mcp.RA117.000470)
Supplement: Supplemental Data [file supp_RA117.000470_133922_0_supp_23978_fzffwf.zip › spectra_annotation/mito_DR_spectra_annotation/221-1-19-1-1-1.pdf]

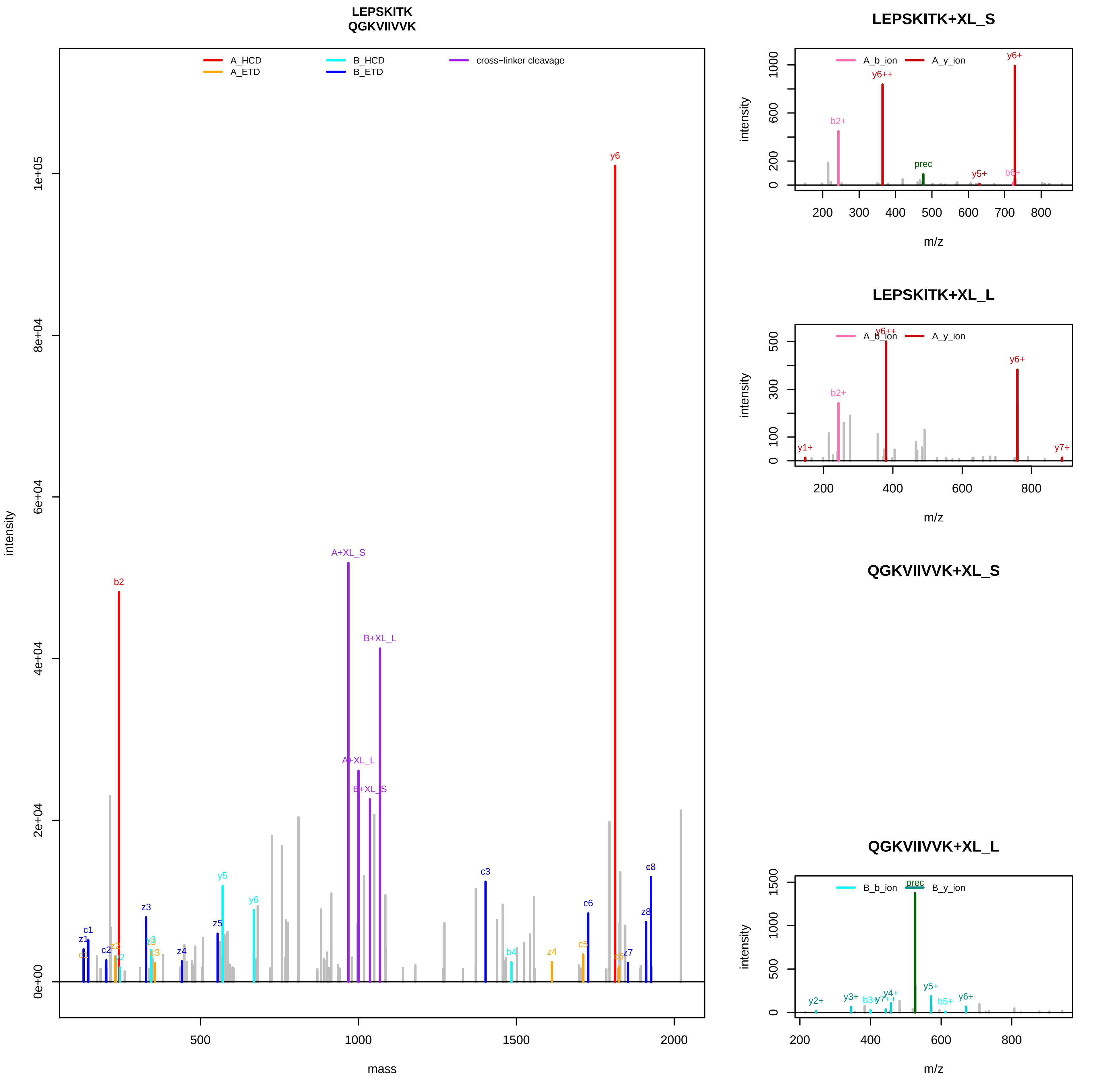

LEPSKITK
QGKVIIVVK
A_HCD
A_ETD
B_HCD
B_ETD
cross−linker cleavage
intensity
mass
0e+00
2e+04
4e+04
6e+04
8e+04
1e+05
500
1000
1500
2000
y6
b2
A+XL_S
B+XL_L
A+XL_L
B+XL_S
c1
z1
c2
z3
y5
y6
z5
z4
c3
b4
z4
c5
c6
z7
z8
c8
LEPSKITK+XL_S
A_b_ion
A_y_ion
y6+
y6++
b2+
prec
y5+
b6+
intensity
m/z
LEPSKITK+XL_L
y6++
b2+
y6+
y1+
y7+
QGKVIIVVK+XL_S
QGKVIIVVK+XL_L
B_b_ion
B_y_ion
prec
y2+
y3+
b3+
y7++
y4+
y5+
b5+
y6+
